# BTB and CNC homology 1 (Bach1) induces lung cancer stem cell phenotypes by stimulating CD44 expression

**DOI:** 10.1186/s12931-021-01918-2

**Published:** 2021-12-23

**Authors:** Pan Jiang, Fan Li, Zilong Liu, Shengyu Hao, Jian Gao, Shanqun Li

**Affiliations:** 1 Department of Nutrition, Zhongshan Hospital, Fudan University, 180 Fenglin Road, Shanghai, China; 2 Department of Pulmonary Medicine, Zhongshan Hospital, Fudan University, 180 Fenglin Road, Shanghai, China

**Keywords:** Bach1, Lung cancer stem cells, CD44, MAPK signaling pathway

## Abstract

**Background:**

Growing evidence suggests that cancer stem cells (CSCs) are responsible for cancer initiation in tumors. Bach1 has been identified to contribute to several tumor progression, including lung cancer. The role of Bach1 in CSCs remains poorly known. Therefore, the function of Bach1 on lung CSCs was focused currently.

**Methods:**

The expression of Bach1, CD133, CD44, Sox2, Nanog and Oct4 mRNA was assessed using Real-Time Quantitative Reverse Transcription PCR (RT-qPCR). Protein expression of Bach1, CD133, CD44, Sox2, Nanog, Oct4, p53, BCL2, BAX, p-p38, p-AKT1, c-Fos and c-Jun protein was analyzed by western blotting. 5-ethynyl-29-deoxyuridine (EdU), colony formation, Flow cytometry analysis and transwell invasion assay were carried out to analyze lung cancer cell proliferation, apoptosis and invasion respectively. Tumor sphere formation assay was utilized to evaluate spheroid capacity. Flow cytometry analysis was carried out to isolate CD133 or CD44 positive lung cancer cells. The relationship between Bach1 and CD44 was verified using ChIP-qPCR and dual-luciferase reporter assay. Xenograft tumor tissues were collected for hematoxylin and eosin (HE) staining and IHC analysis to evaluate histology and Ki-67.

**Results:**

The ratio of CD44 + CSCs from A549 and SPC-A1 cells were significantly enriched. Tumor growth of CD44 + CSCs was obviously suppressed in vivo compared to CD44− CSCs. Bach1 expression was obviously increased in CD44 + CSCs. Then, via using the in vitro experiment, it was observed that CSCs proliferation and invasion were greatly reduced by the down-regulation of Bach1 while cell apoptosis was triggered by knockdown of Bach1. Loss of Bach1 was able to repress tumor-sphere formation and tumor-initiating CSC markers. A repression of CSCs growth and metastasis of shRNA-Bach1 was confirmed using xenograft models and caudal vein injection. The direct interaction between Bach1 and CD44 was confirmed by ChIP-qPCR and dual-luciferase reporter assay. Furthermore, mitogen-activated protein kinases (MAPK) signaling pathway was selected and we proved the effects of Bach1 on lung CSCs were associated with the activation of the MAPK pathway. As manifested, loss of Bach1 was able to repress p-p38, p-AKT1, c-Fos, c-Jun protein levels in lung CSCs. Inhibition of MAPK signaling remarkably restrained lung CSCs growth and CSCs properties induced by Bach1 overexpression.

**Conclusion:**

In summary, we imply that Bach1 demonstrates great potential for the treatment of lung cancer metastasis and recurrence via activating CD44 and MPAK signaling.

## Background

Lung cancer is a malignant tumor with a high rate of morbidity and mortality worldwide. Previous studies have reported that non-small cell lung cancer (NSCLC) accounts for almost 85% of lung cancer cases [1]. The main reason for treatment failure and mortality in NSCLC is cancer cell invasion and metastasis [2, 3]. Although it has been confirmed that multiple tumor-related genes are involved in the modulation of NSCLC development, the detailed molecular mechanism of NSCLC remains unclear.

Recently, it has been reported that there is a small group of cells within tumors that can play an important role in cancer resistance and participate in tumor progression and metastasis, hence are called cancer stem cells [4]. It has been reported that leukemia stem cells were the first CSCs discovered [5]. Cancer stem cells are widely recognized in many types of cancers, such as lung cancer, liver cancer, and pancreatic cancer [6–8]. Lung CSCs are critical for lung cancer metastasis and drug resistance. For example, CD44 is functionally important for activating lung CSCs by regulating the Wnt/β-catenin-FoxM1-Twist pathway [9]. Previously, we reported that epigallocatechin-3-gallate (EGCG) represses CSC-like properties by modulating miR-485 and CD44 in cisplatin-resistant A549 cells [10]. Nuclear-enriched abundant transcript 1 (NEAT1) induces CSC-like traits in lung cancer cells by activating Wnt signaling [11]. Therefore, targeting lung CSCs may be useful for lung cancer therapy.

Bach1 is a crucial transcriptional factor that plays an important role in oxidative stress, the cell cycle, hematopoiesis, and immunity [12–14]. Bach1 has been shown to function as an inducer of metastatic genes in breast cancer, including CXCR4 and MMP1 [15, 16]. Higher Bach1 levels indicate a higher risk of human cancers, such as colorectal cancer, glioblastoma, and lung cancer [17–19]. Bach1 stabilization by antioxidants can induce lung cancer [20]. However, the detailed function of Bach1 in lung cancer stem cells remains unclear.

In this study, we investigated the role of Bach1 in lung cancer. Bach1 is highly expressed in lung CSCs and its knockdown repressed lung CSC growth and metastasis. In addition, Bach1 interacts with CD44 and contributes to lung CSC migration and invasion. Bach1 also activates the expression of genes related to the MAPK signaling pathway. Therefore, our findings indicate that Bach1 can serve as an important transcriptional regulator of lung CSC development. The investigation was conducted to develop promising treatment strategies and improve the effectiveness of lung CSCs.

## Materials and methods

### Cell culture

A549, SPC-A1 and H1299 cells were obtained from the American Type Culture Collection (Manassas, VA, USA). A549, SPC-A1 and H1299 cells were maintained at 37 °C in 5% CO_2_-humidified air in RPMI 1640 medium containing 10% FBS (Sigma-Aldrich, St. Louis, MO, USA), 100 U/mL penicillin, and 100 mg/mL streptomycin/penicillin. CD44 + cells were isolated using the CD44 microbead isolation kit (Miltenyi Biotec, Auburn, CA, USA).

### 5-Eyhynyl-2′-deoxyuridine (EdU) staining assay

Cell proliferation was evaluated via EdU staining using a Cell-Light™ EdU Apollo^®^ 488 In Vitro Imaging Kit (RIBOBIO, Guangzhou, China). Briefly, cells were grown in 96-well plates and cultured to the normal growth stage. EdU labeling was carried out using 100 μL of reagent A. Cells were fixed using 4% paraformaldehyde (PFA) solution and eluted using Triton X-100, followed by Apollo and DAPI staining.

### Cell transfection

Lentiviral vectors of Bach1 and Bach1 shRNA were purchased from GenePharma (Shanghai, China). Lung cancer cells were transduced with LV-Bach1 or LV-shBach1 in the presence of 8 µg/mL polybrene (GenePharma, Shanghai, China) for 48 h. Afterwards, a selection procedure using 10 µg/mL blasticidin for 72 h was performed. Surviving cells were collected and seeded into six-well plates.

### Colony formation

Cells were grown at a density of 500 cells per well of a 6-well plate and maintained in RPMI 1640 medium supplemented with 10% FBS at 37 °C in a humidified atmosphere with 5% CO_2_. After two weeks, the cells were fixed by 4% PFA, and were stained with 0.1% crystal violet.

### Cell invasion assay

Cell invasion was assessed using a Transwell chamber (8-μm pore size; Corning Co., Corning, USA) with Matrigel. At 48 h after transfection, cells were grown in the upper chamber with 10 μg/mL Matrigel. Culture medium containing 10% fetal bovine serum (FBS) was added to the lower chamber. After 48 h, the cells that migrated across the membrane were fixed using methanol, stained with 0.1% crystal violet, and counted under a microscope.

### Flow cytometric analysis

For CD44 + cell analyses, cells were incubated with fluorescence-conjugated monoclonal antibodies against human CD44 (BD Biosciences, Franklin Lakes, NJ, USA). The samples were then analyzed using a FACS Aria III (BD Biosciences, Franklin Lakes, NJ, USA).

### Western blot analysis

Total protein was isolated using a radioimmunoprecipitation assay lysis buffer. The extracted proteins were subjected to 10% SDS-PAGE and transferred to polyvinylidene fluoride membranes. The membranes were blocked with 5% milk and incubated with the primary antibodies at 4 °C overnight, followed by incubation with the corresponding horseradish peroxidase-conjugated secondary antibodies. The signals were detected using an enhanced chemiluminescence detection kit (Cell Signaling Technology, Danvers, MA, USA). The primary antibodies used were antibodies against Bach1 (1:1500, ab180853), CD44 (1:1500, ab189524), Sox2 (1:1500, ab92494), Nanog (1:1500, ab109250), Oct4 (1:1500, ab181557), p53 (1:1500, ab26), BCL2 (1:1500, ab32124), BAX (1:1500, ab32503), p-p38 MAPK and total p38 MAPK (Cell Signaling Technology, Boston, MA, USA), p-AKT1 (1:1500, phospho S473, ab81283), AKT1 (1:1500, ab28422), c-Fos (1:1500, ab190289), c-Jun (1:1500, ab32137 and GAPDH (1:1500, ab8245),all of which were obtained from Abcam.

### qRT-PCR

Total RNA was isolated using TRIzol reagent (Invitrogen). Reverse transcription was performed using the PrimeScript RT Reagent Kit (Takara, Dalian, China). qRT-PCR was conducted using the SYBR Prime Script RT-PCR Kit (Takara). mRNA levels were calculated using the 2^−ΔΔCt^ method [21]. The primers used are listed in Table 1.

**Table 1:** Primers used for real-time PCR

Genes	Forward (5′–3′)	Reverse (5′–3′)
GAPDH	GCACCGTCAAGGCTGAGAAC	TGGTGAAGACGCCAGTGGA
CD44 Sox2 Nanog Oct4 Bach1	GCATTGCAGTCAACAGTCGAAGA GACAGTTACGCGCACATGAA GTGATTTGTGGGCCTGA AGA GGTATTCAGCCAAACGA CCA GAACAGGGCTACTCGCAAAG	CCTTGTTCACCAAATGCACCA TAGGTCTG CGAGCTGGTCAT ACACAGCTGGGTGGAAGAGA CACACTCGGACCACATCCTT AAAGG GCAGTTGACGGAAC

### CD44 promoter analysis

Briefly, cells were transfected with Bach1-FLAG or a control plasmid. After 24 h, cells were transfected with a β-galactosidase plasmid and the wild-type or a mutated version of the CD44 promoter plasmid or the pGL3-basic luciferase reporter plasmid. Luciferase activity was determined using a Luciferase Assay Kit (Promega, Madison, WI, USA). β-galactosidase activity was also evaluated. The relative Luc activity was recorded as the ratio of Luc/β-gal activity.

### Tumor spheroid formation assay

Cells were maintained in serum-free DMEM/F12 medium supplemented with 20 ng/mL epidermal growth factor, 20 ng/mL basic fibroblast growth factor, and B27. Afterwards, the cells were plated onto an ultra-low-attachment 24-well plate at a density of 5000 cells per well. Tumorsphere formation was photographed using a light microscope (Nikon, Japan).

### Chromatin immunoprecipitation (ChIP) assay

Cells were grown in a 10-cm tissue culture dish. The next day, they were cross-linked with 1% formaldehyde for 10 min, followed by genomic DNA fragmentation. The chromatin fragments were immunoprecipitated with 5 μg of the antibody against Bach1 (R&D Systems, Minneapolis, MN, USA) or an antibody against CD44 (R&D Systems). DNA extraction was performed using a Qiagen Purification kit. Subsequently, real-time PCR analysis was conducted using primers amplifying the promoters of Sox2, Nanog, and Oct4.

### Immunohistochemical (IHC) staining

Tissue sections obtained from xenografts were incubated with rabbit polyclonal anti-Ki-67 antibody at 4 °C overnight. Normal rabbit serum was used as a negative control. The cells were then incubated with a horseradish peroxidase-conjugated anti-rabbit secondary antibody (Santa Cruz, CA, USA) at 37 °C for 1 h. A diaminobenzidine substrate kit (Vector Laboratories, Burlingame, CA, USA) was used to develop the signals.

### Xenograft experiments

All animal studies were approved by the Ethics Committee of the Experimental Research at Zhongshan Hospital, Fudan University. Six-week-old nude mice (six mice per group, grouped as CD44+, CD44− LV-NC, and LV-shBach1) were injected subcutaneously into the bilateral flank area with 5 × 10^6^ cells in 200 μL PBS mixed with 100 μL of Matrigel. Tumor volume was recorded every three days. Then, the mice were sacrificed to harvest the tumor tissues for hematoxylin and eosin (HE) staining or IHC analysis. To perform the in vivo tumor metastasis assay, six nude mice per group were injected via caudal vein with 5 × 10^6^ cells in 200 μL of PBS. Lung tissues were observed.

### Statistical analysis

Data are presented as the mean ± standard error of at least three independent assays. SPSS 20.0 and GraphPad Prism v. 6.0 were used for statistical analysis. Two-group comparisons were conducted using Student’s t-test. One-way ANOVA was used to compare multiple groups. Statistical significance was set at P < 0.05.

## Results

### Lung CSCs were successfully enriched from A549 and SPC-A1 cells

**Fig. 1:**
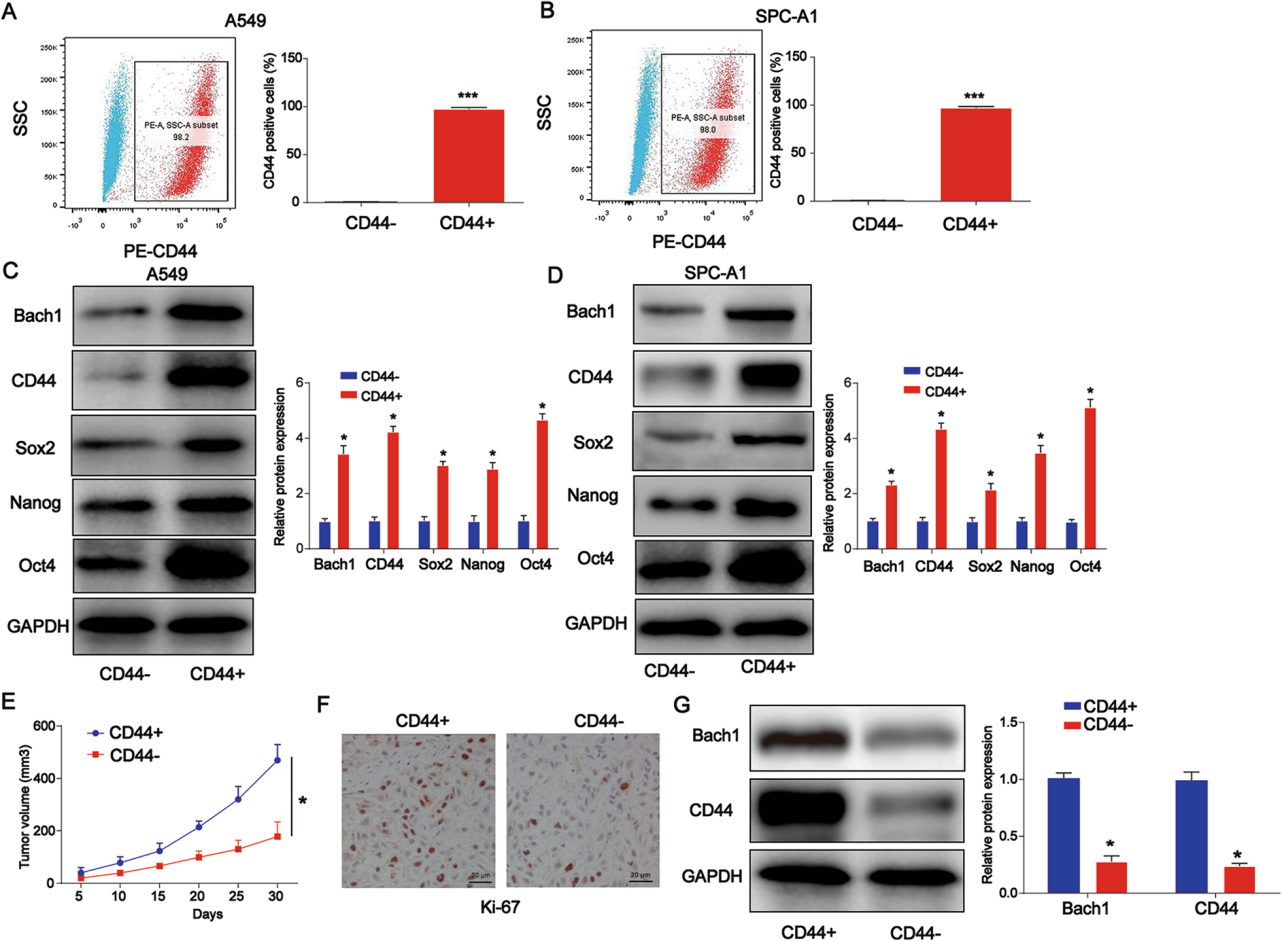
Lung CSCs were successfully enriched from A549 and SPC-A1 cells. **A**, **B** Flow cytometric analysis results demonstrating the percentage of tumorigenic CD44+ A549 and SPC-A1 cells. **C**, **D** CD44+ A549 and SPC-A1 CSCs were analyzed using anti-Bach1, anti-CD44, anti-Sox2, anti-Nanog and anti-Oct4 antibodies using western blotting. **E** Tumor volume was shown in the two different groups. Mice were injected with CD44+ A549 and CD44− cells. **F** Ki-67-positive cell ratios. **G** Bach1 protein expression was evaluated using IHC western blot analysis. *P < 0.05, ***P < 0.001

Recently, CD44 has been identified as a tumor-initiating CSC marker in various cancers. We isolated CD44+ lung cancer cells using flow cytometric analysis as shown in Fig. 1A, B (*P* < 0.001). As shown in Fig. 1C, D (*P* < 0.05), Bach1, CD44, Sox2, Nanog, and Oct4 protein expression was significantly increased in CD44 + A549 and SPC-A1 CSCs as evaluated using western blotting. We then confirmed the tumor growth of lung CSCs in xenografts. Mice were injected with CD44+ A549 and CD44− cells. As shown in Fig. 1E *(P* < *0.05)*, tumor volume was decreased in the CD44− mice group compared to the CD44+ group. As shown in Fig. 1F, the Ki-67 positive cell ratio was markedly increased in the CD44+ group. In contrast, as shown in Fig. 1G, Bach1 and CD44 protein expression was greatly elevated in the mice in the CD44+ group *(P* < *0.05)*.

### Knockdown of Bach1 inhibited lung CSC proliferation, enhanced cell apoptosis, and repressed cell invasion

**Fig. 2:**
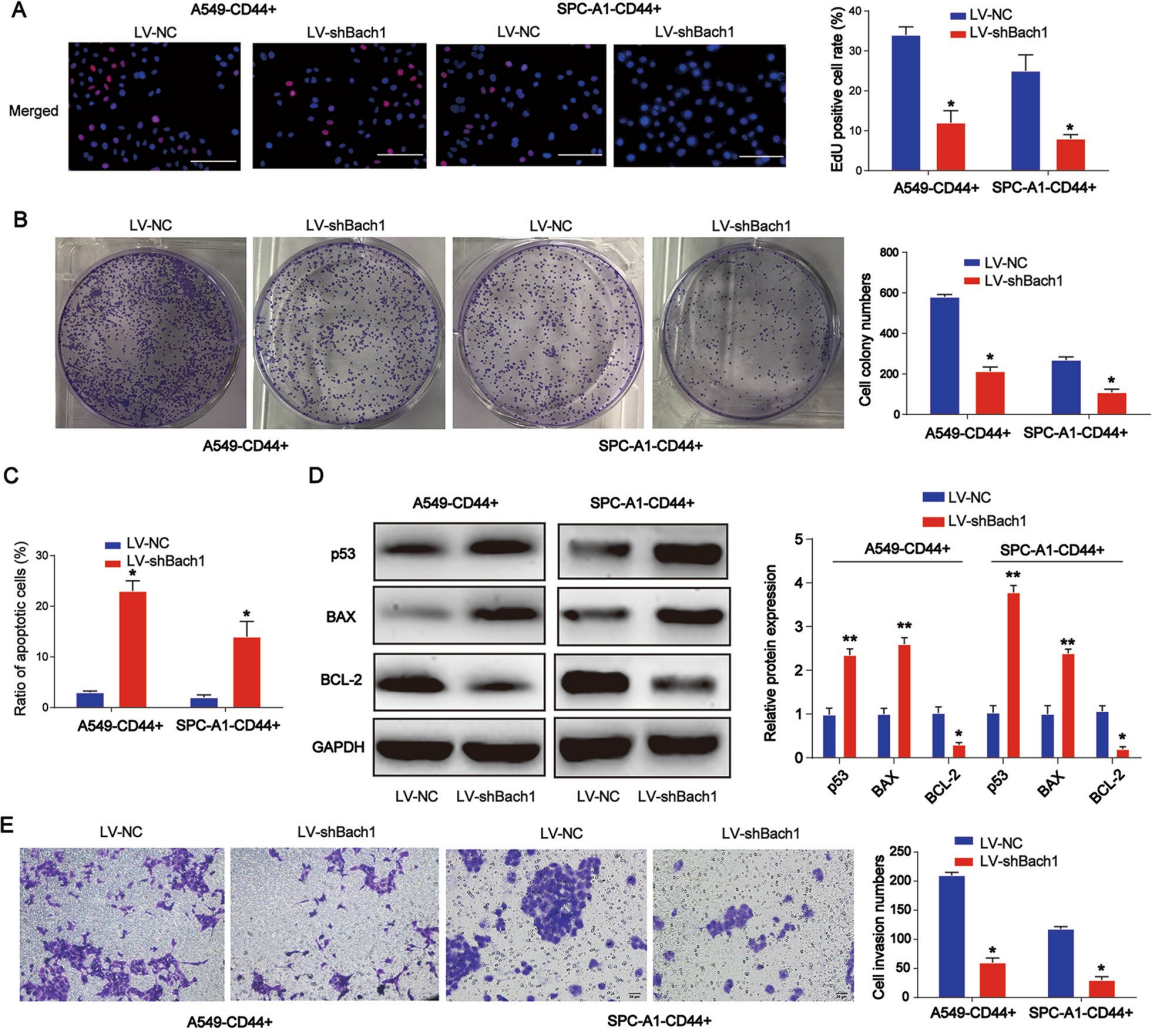
Role of Bach1 in lung CSC proliferation, apoptosis, and invasion. CD44 + cells were cultured at 70% confluency and transfected with LV-shBach1 for 48 h. **A** The EdU assay was performed 48 h post-transfection to determine cell proliferation. **B** A colony formation assay was performed to assess cell colony formation capacity. **C** Flow cytometry was utilized to detect cell apoptosis. **D** p53, BCL2 and BAX protein expression in lung CSCs. **E** A Transwell invasion assay was performed to determine cell invasion ability. *P < 0.05; **P < 0.01

Moreover, to study the potential role of Bach1 in CSC growth, CD44+ cells were transfected with LV-shBach1 for 48 h. The EdU assay was performed 48 h post-transfection and it was observed that cell proliferation was repressed upon treatment with LV-shBach1, as shown in Fig. 2A (*P* < 0.05). Figure 2B (*P* < 0.05) shows that cell colony formation capacity was also reduced with the loss of Bach1. In addition, apoptosis was triggered by LV-shBach1, as shown in Fig. 2C (*P* < 0.05). In Fig. 2D, we checked apoptosis related gene panels including p53, BCL2 and BAX. We found that BCL2 protein expression were reduced by Bach1 shRNA while BAX and p53 were induced by Bach1 shRNA. The Transwell invasion assay showed that cell invasion ability was suppressed by a lack of Bach1 (Fig. 2E, P < 0.05).

### Knockdown of Bach1 restrained lung CSC properties

**Fig. 3:**
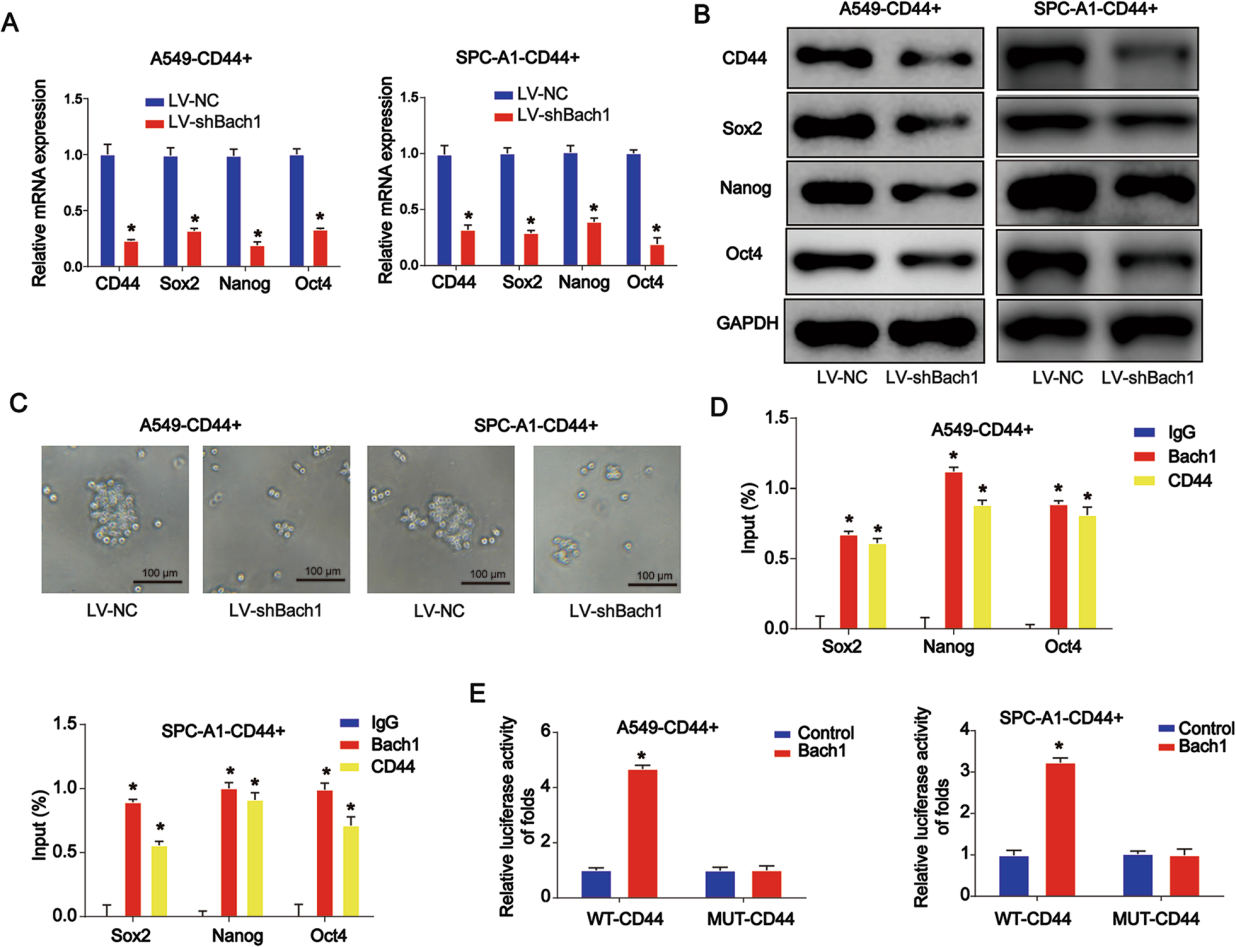
Role of Bach1 in the determination of lung CSC properties. **A** CD44, Sox2, Nanog, and Oct4 mRNA expression in lung CSCs. **B** CD44, Sox2, Nanog, and Oct4 protein expression in lung CSCs. **C** A tumor spheroid assay was carried out to evaluate cell sphere formation ability. **D** ChIP-qPCR was conducted using anti-Bach1 or anti-CD44 antibodies. **E** Analysis of CD44 promoter activity. Cells were transfected with a Bach1-FLAG plasmid for 24 h. *P < 0.05

Next, we proved that CD44, Sox2, Nanog, and Oct4 mRNA and protein expression in lung CSCs was greatly inhibited by LV-shBach1 (Fig. 3A, B, P < 0.05). As shown in Fig. 3C (*P* < 0.05), the tumor spheroid assay indicated that cell sphere formation was remarkably reduced by the loss of Bach1. ChIP-qPCR was also performed (Fig. 3D, P < 0.05) revealing that Bach1 and CD44 co-occupied the promoters of CSC-related genes, as determined using anti-Bach1 or anti-CD44 antibodies. Furthermore, overexpression of Bach1 significantly increased the luciferase activity of the CD44 promoter (Fig. 3E, P < 0.05).

### Bach1 loss repressed the tumor growth and tumor metastasis of lung CSCs

**Fig. 4:**
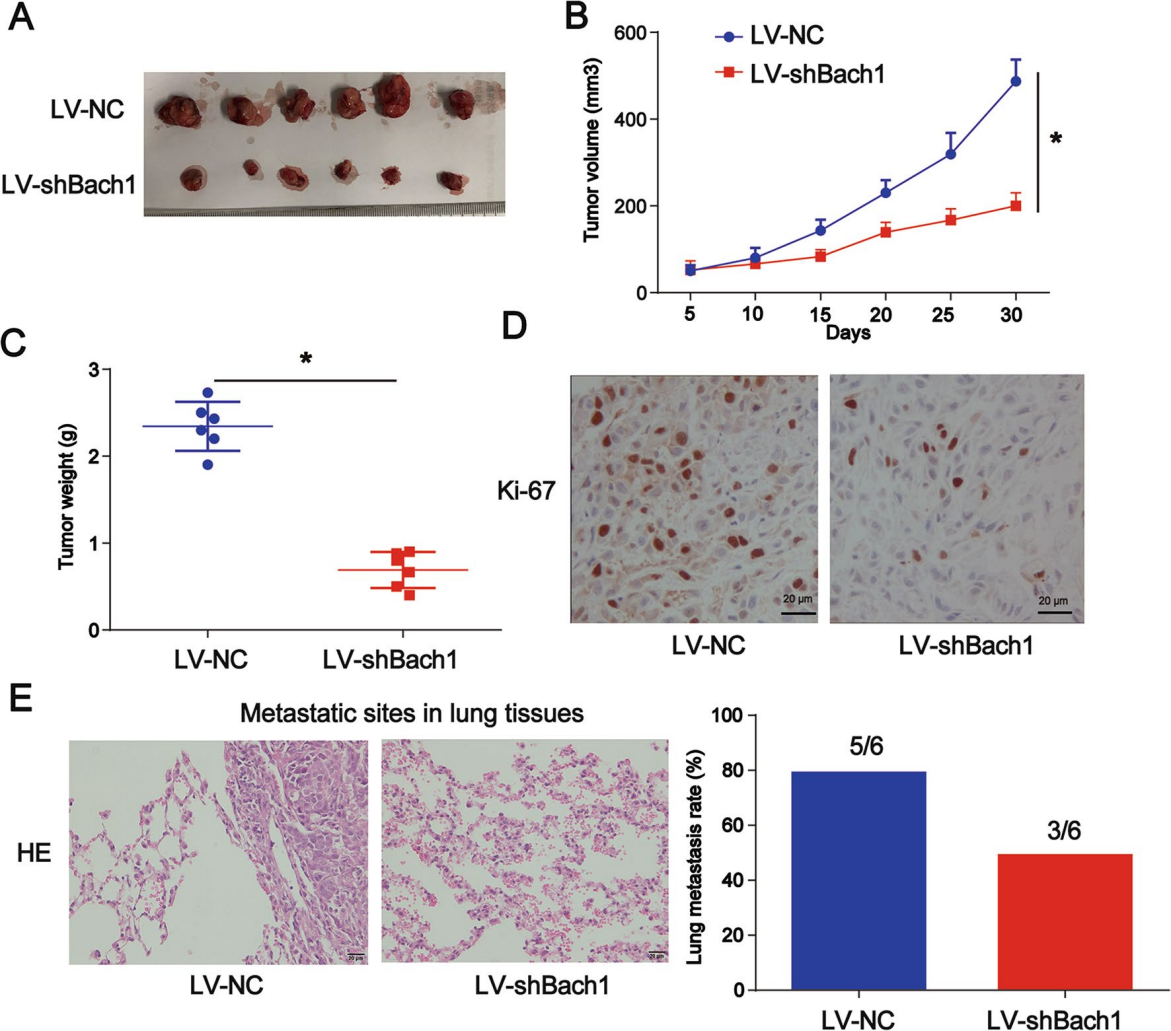
Role of Bach1 on the growth and metastasis of lung CSCs. **A** PET-CT was utilized to observe the tumor growth of lung CSCs in xenografts. **B** Tumor volume change over time. **C** Tumor weight. **D** IHC staining of Ki-67. **E** Loss of Bach1 inhibited the metastasis of lung CSCs in xenografts as evaluated using the HE assay. HE-stained metastasized lung tissues from mice are shown. *P < 0.05

Mice were injected with CD44 + A549 cells transfected with or without Bach1. It was revealed that the tumor growth of lung CSCs in xenografts was significantly reduced after treatment with LV-shBach1 (Fig. 4A). As shown in Fig. 4B, C (*P* < 0.05), tumor volume and weight were both decreased with the loss of Bach1 in vivo. IHC staining for Ki-67 is shown in Fig. 4D. To carry out the in vivo tumor metastasis assay, six nude mice per group were injected via caudal vein with 5 × 10^6^ CD44 + A549 cells transfected with or without Bach1cells in 200 μL of PBS. As shown in Fig. 4E (*P* < 0.05), the loss of Bach1 inhibited the metastasis of lung CSCs in xenografts as evaluated using the HE assay.

### Relation of MAPK signaling with lung CSCs

**Fig. 5:**
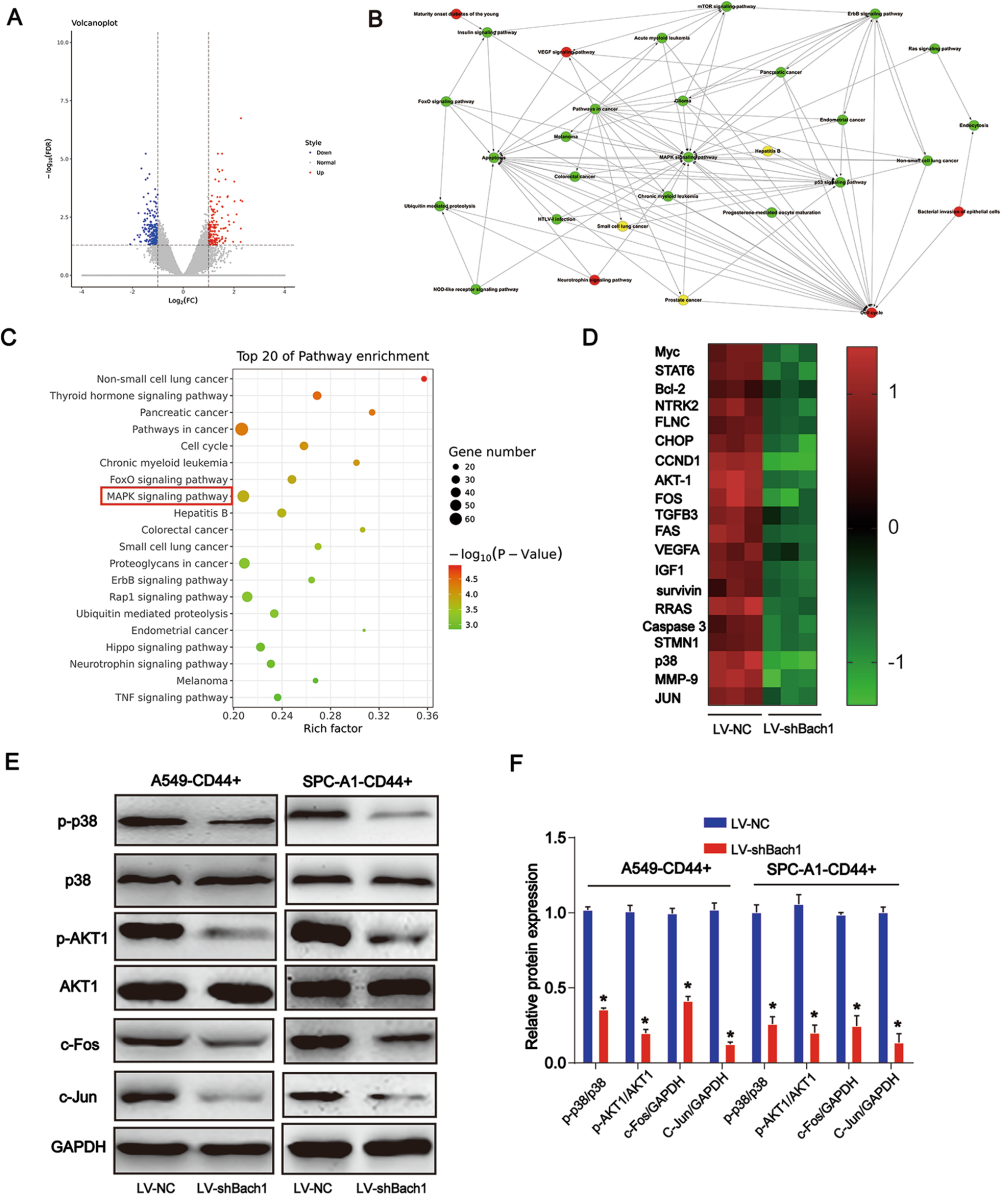
Relationship of MAPK signaling and lung CSCs. Microarray analysis was applied to screen the mRNAs which can be regulated by Bach1. **A** Volcano plot of the data showing altered gene expression in A549 CSCs transfected with or without LV-shBach1. **B**, **C** KEGG pathway analysis revealed the signaling pathways potentially involved in Bach1-mediated functions. **D** Top 20 genes involved in the MAPK signaling pathway. **E**, **F** p-p38, p-AKT1, c-Fos and c-Jun protein expression

Moreover, microarray analysis was performed to explore the underlying molecular mechanisms of Bach1. Gene expression in A549 CSCs transfected with LV-NC or LV-shBach1 was analyzed using RNA sequencing. A volcano plot of the data showing altered gene expression in A549 CSCs transfected with or without LV-shBach1 is presented in Fig. 5A. Gene Ontology (GO) analysis and Kyoto Encyclopedia of Genes and Genomes (KEGG) pathway indicated the functions and signaling pathways associated with the target genes [22]. KEGG pathway analysis indicated the close association between Bach1 and the MAPK signaling pathway, as shown in Fig. 5B, C. Figure 5D shows the top 20 genes involved in the MAPK signaling pathway. We proved that p-p38, p-AKT1, c-Fos and c-Jun protein expression was significantly decreased upon treatment with LV-shBach1 (Fig. 5E, F, P < 0.05).

### Inhibitors of MAPK signaling repressed CD44 + CSC characteristics

**Fig. 6:**
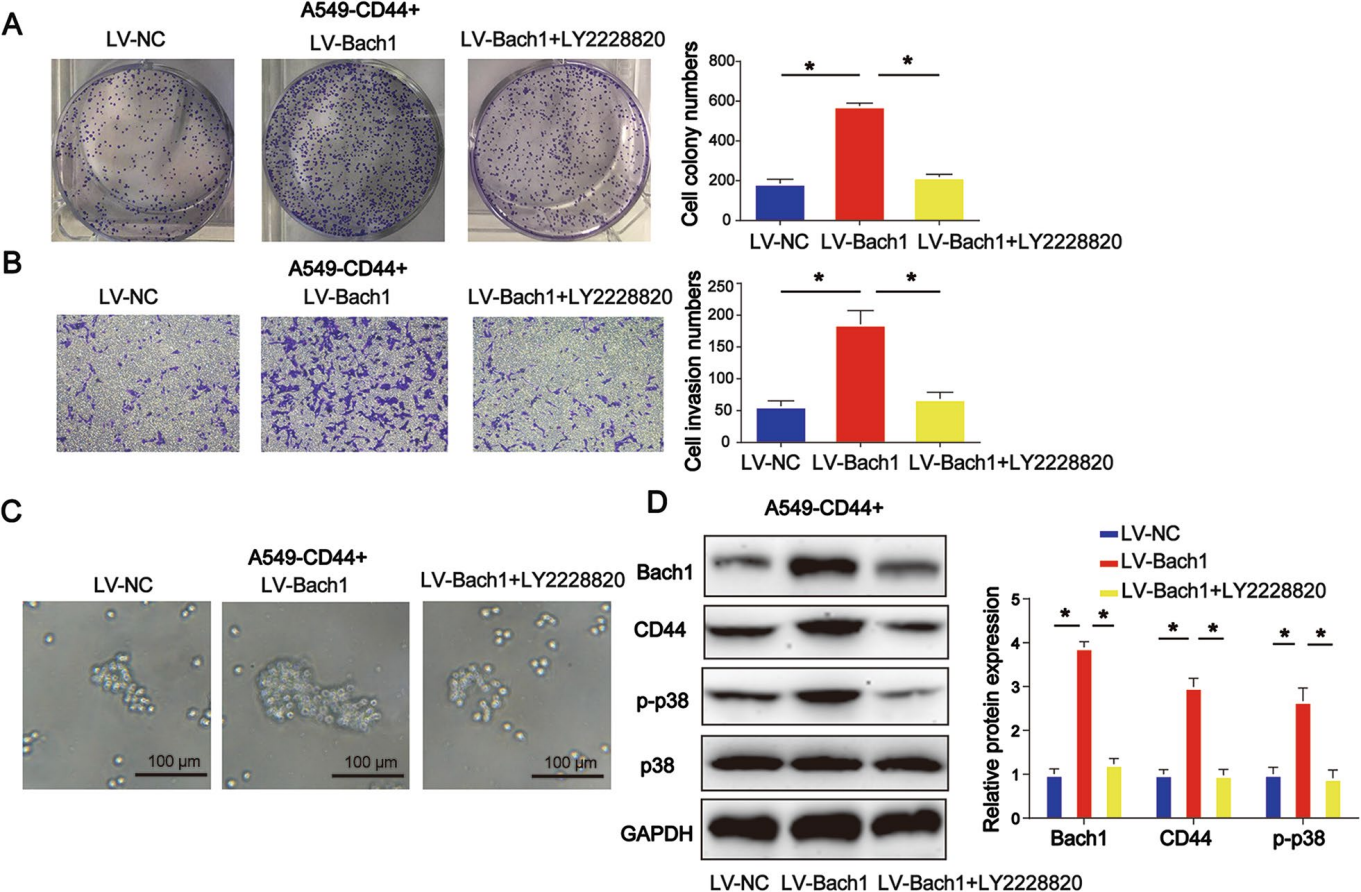
Inhibitors of MAPK signaling repressed CD44 + CSC characteristics. **A** Colony formation capacity. **B** A Transwell invasion assay was carried out to test cell invasion ability. **C** A tumor spheroid assay was performed to evaluate cell sphere formation ability. **D** Bach1, CD44, and p-p-38 protein expression in A549 CSCs. *P < 0.05

LY2228820 is commonly used as a MAPK inhibitor. Results of the colony formation assay implied that A549 CSC colony formation capacity was induced upon overexpression of Bach1, while LY2228820 repressed this process (Fig. 6A, P < 0.05). Consistently, cell invasion ability was also reduced by MAPK inhibitors (Fig. 6B, P < 0.05). Additionally, results of the tumor spheroid assay revealed that LY2228820 suppressed cell sphere formation, as shown in Fig. 6C. As shown in Fig. 6D (*P* < 0.05), Bach1, CD44, and p-p-38 protein expression triggered by LV-Bach1 was greatly reduced by LY2228820 in A549 CSCs.

## Discussion

Patients with lung cancer whose tumors have undergone metastasis have low survival rates [23]. Mounting evidence indicates that CSCs are mainly responsible for cancer aggressiveness, drug resistance, and tumor relapse. Studies of genes involved in lung CSCs are critical for understanding their therapeutic potential. In the present study, the effect of Bach1 on the progression of lung CSCs was determined. To our knowledge, this is the first study to report the potential mechanism of Bach1 in lung CSCs. Loss of Bach1 markedly reduced growth, invasion, and CSC-like properties in lung CSCs in vivo and in vitro by activating CD44 expression. In addition, we found that the knockdown of Bach1 inactivated the MAPK signaling pathway.

Accumulating evidence has shown that Bach1 contributes to tumor metastasis [15]. For example, knockdown of the Bach1 gene suppresses the invasion of breast cancer cells by targeting the expression levels of matrix metalloproteinase-9 and CXCR4 receptor [24]. Bach1 silencing significantly inhibited the migration of colon cancer cells by inhibiting metastasis-related genes [25]. It has been shown that Bach1 can form a complex with MAFG and the DNA methyltransferase DNMT3B, which resulted in the repression of tumor suppressor genes [26, 27]. It has been reported that Bach1 can interact with Nanog, Sox2, and Oct4, thereby maintaining stem cell identity and self-renewal of human embryonic stem cells [28]. In the present study, we observed that Bach1 was highly expressed in lung CSCs. The upregulation of Bach1 in lung CSCs may be associated with an increase in the expression of CSC-associated genes. However, the specific mechanisms underlying the elevation of Bach1 in lung CSCs have yet to be investigated. Previously, we reported that chronic intermittent hypoxia induced lung cancer stem cell-like properties through enhancing Bach1 expression [29]. In addition, we would further check the detailed mechanism that Bach1 induces lung tumor growth and reduces lung cancer cell death. For example, as reported, Bach1 can mediate suppression of p53 is inhibited by p19(ARF) independently of MDM2 [30]. In glioblastoma, Bach1 promotes temozolomide resistance through antagonizing the function of p53 [17].

Emerging roles of CD44 have been demonstrated in CSCs and show that it may be a promising biomarker for different types of cancers [31]. Mounting evidence has shown that CD44 is a crucial CSC marker and is critical in regulating cancer cell self-renewal, tumor initiation, and metastasis. Therefore, CD44 is widely used to enrich CSCs through fluorescence-activated cell sorting of dissociated single cells [32]. It has been reported that lung cancer cells expressing CD44 have enriched CSC properties [33]. In the present study, single cells were isolated from A549 and SPC-A1 tumor cell cultures. We found that Bach1 expression was increased in lung CSCs and was positively correlated with CSC-related gene expression in lung CSCs. In addition, we proved that the loss of Bach1 greatly reduced the expression of the CSC-related genes Sox2, Nanog, and Oct4; however, the relationship between CD44 and Bach1 remains unknown. A positive correlation between CD44 expression and Bach1 levels has been observed in lung CSCs. In addition, the loss of Bach1 was able to repress CD44 expression. Subsequently, we confirmed that CD44 is a binding partner of Bach1 in lung CSCs. More studies are needed to explore the correlation between CD44 and Bach1 in lung CSCs.

The MAPK signaling pathway is an important signaling cascade that can promote cell survival and differentiation [34–36]. Activation of the MAPK signaling pathway plays a positive role in tumor metastasis in numerous human malignancies, including lung cancer [37]. The lncRNA TUC338 can promote the invasion of lung cancer through the MAPK pathway [38]. In addition, lncRNA SNHG12 triggers multidrug resistance by activating the MAPK/Slug pathway in lung cancer [39]. Further studies have revealed that CD44 acts as an ERK-dependent downstream effector of serglycin, which can activate the MAPK/β-catenin axis to induce CD44 receptor expression in nasopharyngeal carcinoma [40]. CD44 can regulate a number of central signaling pathways, including the PI3K/AKT, Rho GTPase, and Ras-MAPK pathways [41]. In our study, we found that Bach1 silencing significantly repressed the MAPK pathway. Hence, we showed the relationship of the MAPK signaling pathway and Bach1 in promoting lung CSC growth and CSC-like properties. We also proved that MAPK inhibitors reversed CSC growth and CSC-like characteristics induced by the overexpression of Bach1. Crosstalk between Raf-MEK-ERK and PI3K-Akt-GSK3β signaling can promote chemoresistance, progression and stemness through regulating the expression of CD44 in oral cancer [42]. However, the downstream target proteins of MAPK signaling that are linked to CD44+ should be explored in a future study. Among MAPK gene panels, p-p38, p-AKT1, c-Fos and c-Jun were evaluated and we found p-p38, p-AKT1, c-Fos and c-Jun protein expression was reduced by loss of Bach1.

In conclusion, these results demonstrated that silencing Bach1 played a critical role in the growth of lung CSCs, suppressing cell proliferation and promoting cell apoptosis via activating CD44 and MAPK pathway. Therefore, our research exerted far-reaching significance in developing promising therapy for lung cancer patients.

## Data Availability

The data that support the findings in this study are available from the corresponding author upon reasonable request.
